# Binomial outcomes in dataset with some clusters of size two: can the dependence of twins be accounted for? A simulation study comparing the reliability of statistical methods based on a dataset of preterm infants

**DOI:** 10.1186/s12874-017-0369-6

**Published:** 2017-07-20

**Authors:** Odile Sauzet, Janet L. Peacock

**Affiliations:** 1 Epidemiology and International Public Health, School of Public Health, Bielefeld University, Bielefeld, Germany; 2 Division of Health and Social Care Research King’s College London and NIHR Biomedical Research Centre at Guy’s and St Thomas’ NHS Foundation Trust and King’s College London, London, UK

**Keywords:** Binomial outcomes, Small clusters, Generalised mixed models, Generalised estimating equations, Perinatal outcomes

## Abstract

**Background:**

The analysis of perinatal outcomes often involves datasets with some multiple births. These are datasets mostly formed of independent observations and a limited number of clusters of size two (twins) and maybe of size three or more. This non-independence needs to be accounted for in the statistical analysis. Using simulated data based on a dataset of preterm infants we have previously investigated the performance of several approaches to the analysis of continuous outcomes in the presence of some clusters of size two. Mixed models have been developed for binomial outcomes but very little is known about their reliability when only a limited number of small clusters are present.

**Methods:**

Using simulated data based on a dataset of preterm infants we investigated the performance of several approaches to the analysis of binomial outcomes in the presence of some clusters of size two. Logistic models, several methods of estimation for the logistic random intercept models and generalised estimating equations were compared.

**Results:**

The presence of even a small percentage of twins means that a logistic regression model will underestimate all parameters but a logistic random intercept model fails to estimate the correlation between siblings if the percentage of twins is too small and will provide similar estimates to logistic regression. The method which seems to provide the best balance between estimation of the standard error and the parameter for any percentage of twins is the generalised estimating equations.

**Conclusions:**

This study has shown that the number of covariates or the level two variance do not necessarily affect the performance of the various methods used to analyse datasets containing twins but when the percentage of small clusters is too small, mixed models cannot capture the dependence between siblings.

## Background

In clustered data the basic assumption that observations are independent is violated within clusters and an adequate statistical analysis must be performed. Various authors explored the issue in the area of randomised controlled trials [1, 2] where various methods have been proposed such as using cluster-level summary measures [3] and mixed models [4]. In cluster trials, the clusters are typically quite large but the number of clusters is relatively small. This situation contrasts strongly with the structure of the data in samples of infants where most observations are independent (i.e. from singleton births) but a few are clustered (i.e. from multiple births). Here we ignore possible higher level of clustering like at hospital level. This is often the case in datasets of preterm infants where there may be as many as 20% multiple births such as in the United Kingdom Oscillation Study (UKOS) [5, 6]. For these reasons, samples of birth data contain mostly clusters of size one, i.e. singleton births, and the remaining observations are in clusters of size two (twins) or more for higher order multiple birth. This therefore produces a mix of data where the majority are independent (i.e. from singletons), but a minority are non-independent (i.e. from the multiple birth siblings). Before methods to control for non independent data were widely available, researchers analysing studies among preterm infants have tended to ignore the non-independence in such data and treated the multiple births as if they were independent observations [7]. Recalling what we already mentioned in [8], researchers have discussed the methods available to deal with clustering in different contexts; Gates adjusted the standard error for a binary outcome in multiples [9], Carlin analysed twins using mixed models and GEE [10], Louis discussed a range of approaches including mixed models and GEEs for analysing studies of repeated pregnancies [11], and Shaffer also compared mixed models and GEEs for continuous and binary outcome models without covariates [12]. In a previous publication [8], we explored the effects of having different proportions of multiples in models with a continuous outcome that included different types of covariates. Our conclusions were that there were no particular threshold in the proportion of multiples from which mixed models would start making a difference. For any percentage of twins, the standard errors were better estimated from mixed models than from a linear regression. There were only very limited problems of convergence.

However, there is only limited evidence available on the effect of not controlling for the dependence of twins for binary outcomes. A comparison of methods of analysis of binomial outcomes by Ananth et al. [13] indicated large differences in values obtained for the standard errors of estimates in a dataset containing only twin pairs. A recent work by Ying et al. [14] looked into at the behaviour of various methods of analysis of binary outcomes in simulated datasets with either limited fixed number of small clusters at 10% or a number of clusters of size two depending on covariate values. Similarly McNeish [15] simulated datasets with small cluster size focusing on the performance of GEE models but did not cover the case where most cluster are of size one. Their conclusions were that all methods compared well but the logistic regression provided biased results for one scenario with sample size 200.

Therefore open questions remain as for what to expect faced with a real dataset containing a realistic percentage of twins when a true binomial outcome needs to be analysed. These questions include how much the percentage of twins matters, the number of covariates, and the size of the effect of covariates and their nature (categorical or continuous) for results to be reliable. Using a similar simulation strategy as in [8] based on a real dataset, we compare parameter estimates and standard errors obtained by three models with several widely available methods of estimation: generalized linear regression (no control for dependence of siblings), generalised linear mixed models and generalized estimating equations. As well as varying the percentage of twins, we also look at the effect of the random intercept variance and of the number of covariates on the reliability of parameter estimates and their standard error as well as the occurrence of non convergence in mixed models. To illustrate this work we surveyed which methods have been used in recently published articles in some high impact journals where datasets of preterm infants were analysed.

## Method

### Survey

A small survey of how twins are accounted for in the analysis of datasets of preterm children was performed in order to illustrate the practice. Three journals were chosen, two general, the British Medical Journal (BMJ) and the Journal of the American Medical Association (JAMA) and one specialist, Pediatrics. The aim of the survey was to record the percentage of twins in the datasets analysed and whether the correlation between siblings was accounted for, and how. The aim was to find 10 studies in the general journals and 20 in the specialist one within the last ten years. Studies in which we felt it was justified to exclude multiple births because multiple birth was a confounding factor, were not considered for the survey.

### Simulation study

We follow a similar simulation strategy as in our previous work [8]. Datasets were simulated using the distributions of variables obtained from the original dataset of the UKOS study [5].

#### Simulated data

Simulation of the covariates: Simulation parameters were obtained from the UKOS dataset including 797 live birth preterm infants among which 190 were from a multiple birth. The two binary outcomes of interest are *death* (before hospital discharge) and *oxygen dependence at 36 weeks post-menstrual age (* *O*_*2*_ *dep)* of which there were 26% and 56% respectively. These outcomes are commonly used as main outcomes in neonatal trials. Only birth-related covariates were considered so that the outcomes were not playing any censoring role in the data. While here death is considered as a binomial outcome in this simulation study, it should be noted that useful information is lost by ignoring the time to death and that a survival analysis should be preferred. The covariates we considered are birthweight (BW, in grams), sex, Apgar score evaluating the physical condition of a newborn shortly after delivery [16], gestational age (in weeks) and smoking status of the mother because they were significant predictors of at least one of the two outcomes of interest.

For each outcome, data were simulated generating four covariates. The continuous covariates were simulated assuming a multivariate normal model with parameters obtained from the original dataset for single children and twins separately using a separate model per set of levels of categorical variables. This strategy will model the dependence of the outcome with the size of the cluster.

We treated Apgar score as continuous but generated values above 12 were replaced by 10 and generated values between 10 and 12 were replaced by 9. In this way we obtained a distribution of scores similar to the original one. Probabilities of all combinations of categorical outcomes were obtained. These, together with means and variance-covariance matrices, are provided as supplementary material.

Given a set of simulated covariates for infant *j*, a 2-level model for the logit of the probability *p*_*ij*_ of *death* and *O*_2_ *dep* of infant *j* in cluster *i* is assumed:

$$\text{logit}(p_{ij})=\beta_{0}+u_{0i}+\beta_{1}BW_{ij}+\beta_{2}SEX_{ij}+{\ldots} $$

where the type and number of covariates varied. The values of regression parameters (*β*s) were obtained for the UKOS dataset using a random intercept model with the Gauss-Hermite method of estimation. Those *β* parameters were similar to those obtained using a MCMC method. The covariates and their *β* values used in the simulations are shown in Table 1 and form the “true parameters” to which other models will be compared.

**Table 1:** Parameters values for the simulation scenarios

Outcome	Dependent variables						
*death*	intercept	bweight (g)	sex (male)	gest. age (w)	Apgar	Variance*	ICC
4 var.	10.70	-0.004	0.410	-0.039	-0.212	0.48	0.13
2 var.	3.037	-0.006	0.598			0.79	0.20
*O*_2_ *dep*	intercept	bweight (g)	sex (male)	gest. age (w)	smoking	Variance*	ICC
4 var.	14.7	-0.0045	0.954	-0.058	0.654	2.80	0.47
2 var.	5.315	-0.006	1.001			2.95	0.48

^*^Random intercept, these values are given for information only, they were not used for the simulations

The value for *death* or *O*_2_ *dep.* are then obtained randomly following a Bernouili model with probability *p*_*ij*_.

The following algorithm was used:

- Obtain the number of singletons *n*_*s*_ and twin pairs *n*_*t*_ given the size of the dataset and the percentage of cluster of size two.
- Obtain *n*_*s*_ and *n*_*t*_ sets of levels for categorical variables using a multinomial random distribution (for outcome *death*, sex only and for outcome *O*_2_ *dep*, sex and smoking).
- Given a combination of levels, the corresponding vector of means and variance-covariance matrix is used to obtain the continuous covariates using a multivariate normal distribution for one singleton child or for the two siblings in the case of twins.
- For each cluster, *u*_0*i*_ is obtained from a normal distribution $N\left (0,\sigma _{u_{0i}}^{2}\right)$.
- Eq. 1 provides the probability of *death* or *O*_2_ *dep*.
- Given this probability, the outcome is obtained using the corresponding binomial distribution.

For each scenario (sample size of cluster, percentage of twins and variance) 5000 simulated datasets were obtained. The fixed percentage of twins ranged from 2 to 20%. This range was chosen to be large enough to show any trend but limited by the necessary time to run the simulations. The number of clusters were 150, 500 and 1000. The random intercept variance $\sigma ^{2}_{u_{0i}}$ were 0.5, 1 and 2. In order to check if the patterns observed with these variances remained with increasing variance, a limited number of simulations were also performed with variance of 4 and 8.

#### Regression models and analysis of results

Once simulated, the datasets were analysed using three models: logistic regression model (logistic regression), logistic random intercept model and generalized estimating equations (GEE). Three methods of estimation were used for the multilevel models: penalised quasi-likelihood (PQL), adaptive Gauss-Hermite (GH) with 5 point per axis [17]. For these R packages glmmPQL, lme4 and geepack were used.

Given a scenario, all parameter estimates for the intercept and covariates in the model, standard errors and when appropriate (logistic random intercept model) the variance component, were collected. If a convergence problem defined as an error message being returned instead of estimated parameters, was reported then “NA”s were collected in order to provide the percentage of non-convergence. For the PQL and GEE methods of estimation, while the model almost always reported estimates, those were, in few cases, of an unlikely magnitude (10^12^). Therefore, we replaced all the parameter estimates given by this method for a dataset in which the estimation of at least one parameter was above 1000 in absolute value by “NA” i.e. reporting non-convergence for the corresponding method of estimation. This value was arbitrary but estimates were either small or very large. Therefore the choice of cut-point did not influence the results.

Assessment of the different methods of analysis included the bias of the estimates for the intercept, birthweight and sex for both outcomes and number of covariates as well as the variance of the intercept for logistic random intercept models, how accurately the standard error measured the variability of the estimates and the overall quality of estimates with the mean squared error.

The relative bias for the conditional models (Logistic regression and and mixed logistic regression) was calculated as the mean value, over all the simulated datasets for a given scenario for which convergence was reached, of

$$\left(\hat\beta-\beta\right)/{\beta}. $$

Where the $\hat \beta $s are the estimated parameters obtained from the simulated data and the *β*s are the parameters used to simulate the data. Therefore a positive bias indicates that on average the estimated parameter overestimated the true value.

In order to obtain the relative bias for the population average model (GEE), as the “true *β* values” are only known for the random intercept model, we used the following approximate relationship between marginal (GEE) and conditional (logistic random intercept model, logistic Regression) parameters (see [18] Chap. 7.4)

$$\beta_{GEE}=\sqrt{\frac{1}{0.346*\sigma_{u0}^{2}+1}}\beta_{logistic regression} $$

where $\sigma _{u0}^{2}$ is the true random intercept variance. While the parameters estimated from a logistic regression model differ from the one estimated from a random intercept model, we assume that they are interpreted in the same way by researchers and therefore we do not use any corrections for them in the calculation of bias.

We also provided the empirical bias with 95% confidence intervals by calculating the difference between a mean estimate over all simulated datasets.

The coverage of the 95% confidence interval was assessed calculating the proportion of simulated datasets for which *β* is included in the confidence interval $[\hat \beta -1.96SE\ ;\ \hat \beta +1.96SE]$ (using for GEE *β*_*GEE*_ instead of *β*). We based this proportion on the simulated datasets for which the estimation algorithm converged.

The overall quality of the parameter estimates is measured with the mean squared error (using for GEE *β*_*GEE*_ instead of *β*).

$$MSE=\text{var}(\hat\beta)+\left(E(\hat\beta)-\beta\right)^{2}. $$

## Results

### Survey

The aim was to obtain 10 studies in the two general journals and 20 in the specialised one. Commencing in 2013, we have been able to survey 38 studies including preterm infants up to 2012 for Pediatrics, 2003 for JAMA, and 2004 for the BMJ. They showed that only about a third (12/38) mentioned the percentage of multiple birth which varied from 10 to 30%. Two studies did remove multiple birth from the analysis without justification (the effects of being preterm were being analysed). Only a fifth (8/38) of studies mentioned controlling for the non independence of sibling in their analysis. The most frequent method was to use robust estimates for standard errors (4/8), two studies used GEEs and one generalised linear mixed models. One study used multiple birth as a covariate.

### Simulation study

#### Convergence

Percentages of cases of non-convergence for each scenario are reported in Table 2 for numbers of clusters of 150 and 500. The patterns of convergence were different for the two outcomes but an effect of the number of clusters was clear in both cases. For PQL and GEE there were scenarios with a percentage of non-convergence above 1% for a number of clusters of 150 whereas the estimation algorithm always converged for sample sizes of 500 or more. For the outcome *death*, the method of estimation GH had percentage of non-convergence in the range of 2 to 19% and 0 to 1% respectively for a number of clusters of 150.

**Table 2:** Percentage of non-convergence for 150 and 500 clusters

Outome/	Method of	Random intercept	Perc. of non-	Random intercept	Perc. of non-
No of clusters	estimation	variance	convergence*	variance	convergence*
		*2 covariates*		*4 covariates*	
*death*	PQL	0.5 to 2	3 to 0%	0.5 to 2	53 to 0%
150	GH	0.5 to 2	0 to 1%	0.5 to 2	0 to 1%
	GEE	0.5 to 2	3 to 1%	0.5 to 2	3 to 1%
*death*	PQL	0.5 to 2	≤ 1%	0.5 to 2	≤ 1%
500	GH	0.5 to 2	0%	0.5 to 2	0%
	GEE	0.5 to 2	≤ 2%	0.5 to 2	≤ 1%
*O*_2_ *dep*	PQL	0.5 to 2	0%	0.5 to 2	18 to 0%
150	GH	0.5	20 to 27%	0.5	0%
		1	19 to 28%	1	0%
		2	18 to 28%	2	0%
	GEE	0.5 to 2	5 to 0%	0.5 to 2	6 to 0%
*O*_2_ *dep*	PQL	0.5 to 2	0%	0.5 to 2	0%
500	GH	0.5	37 to 48	0.5	≤ 1%
		1	35 to 46%	1	≤ 1%
		2	34 to 46%	2	≤ 1%
	GEE	0.5 to 2	0%	0.5 to 2	≤ 1%

^*^for percentage of twins ranging from 2 to 20%

There was also an effect of the percentage of clusters of size two (twins) independent of the sample size effect. For PQL and GEE the percentage of non-convergence decreased with the percentage of clusters (e.g. *death*: 2 covariates, number of cluster=150 GEE model, non-convergence of 3% for 2% of twins and 0% for 20% of twins, variance=2) whereas the percentage of non-convergence increased for GH with the percentage of twins.

For GEE and PQL, the effect of the cluster level variance seems very small but for GH the effect is that increasing the variance does increase the percentage of non-convergence.

The effect of the number of covariates depends on the model coefficients as far as GH are concerned. While for the outcome *death* there were slightly less instances of non-convergence in models with two covariates than with four covariates for GEE and PQL, the picture is different for GH with either the same low instance or much more for two covariates than with 4.

Having two simulated outcomes showed the potential differences that model parameters can have on convergence. For the outcome *death* the percentage of non-convergence were very small for GH. For the outcome *O*_2_ *dep*, models with two covariates had a percentage of non-convergence for the method GH which increased with the number of clusters and reached the range of 55-64% for 1000 clusters and variance of 0.5 (data not shown). Such problems were not seen for the model with four covariates.

### Estimation of regression parameters

**Fig. 1:**
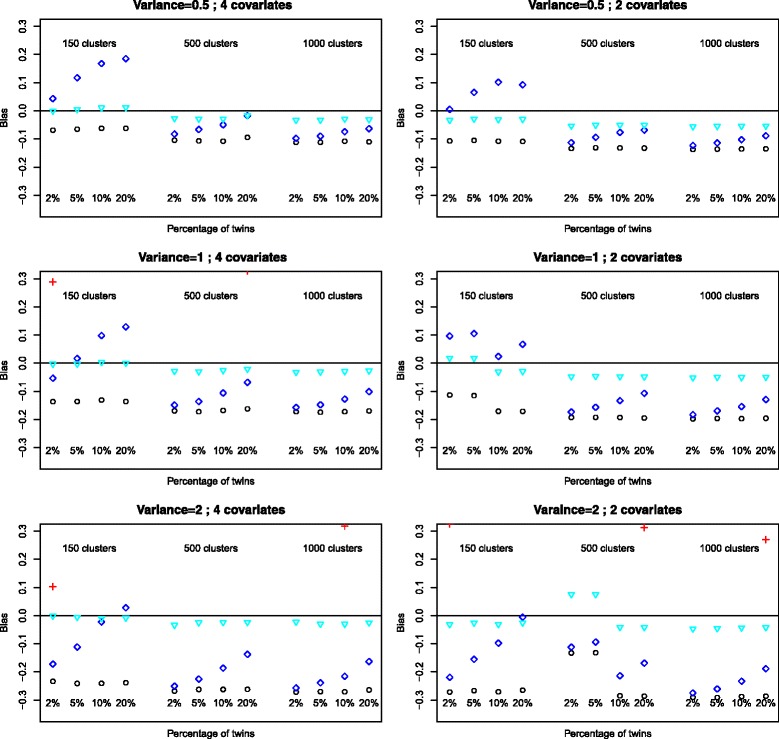
Bias of estimates for the variable *Birthweight*, outcome *death*. Logistic: ∘, random intercept, PQL: +, random intercept Gauss-Hermite: ♢, GEE: $\triangledown $

**Fig. 2:**
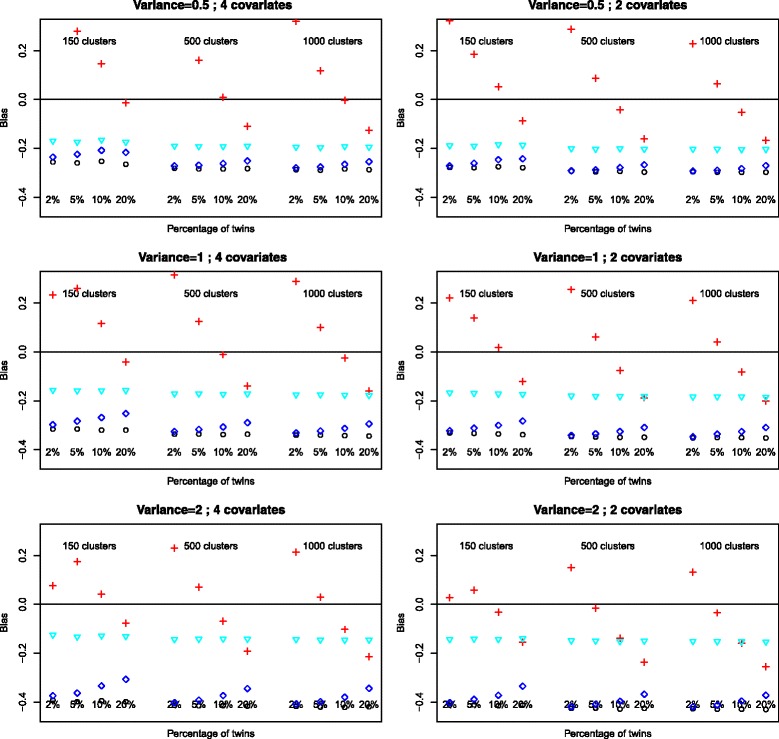
Bias of estimates for the variable *Birthweight*, outcome *O*_2_ *dependence at 36 weeks*. Logistic: ∘, random intercept, PQL: +, random intercept Gauss-Hermite: ♢, GEE: $\triangledown $

Given a simulation model the patterns of bias for the parameter estimates of intercept, birthweight and sex were similar showing that each method of estimation provided similarly good or poor estimates for all parameters. There is no distinction to be made between continuous and categorical covariates. The relative bias for the parameter of birthweight are presented in Figs. 1 and 2.

The relative bias for GEE estimates was not affected by sample size, percentage of twins, number of covariates or level two variance and was consistently over all scenarios the model which provided the least biased estimates. The bias for the logistic regression was not affected by sample size, percentage of twins or number of covariates but increased with increasing random intercept variance. The PQL method of estimation provides extremely unreliable estimates with very variable bias. The method of estimation GH provides estimates with equal or similar bias in most situations we have simulated. The amount of bias decreases with increasing percentage of twins but increases with increasing random intercept variance. There is a similar increase in bias for the logistic regression with the increase in random intercept variance.

The empirical bias with 95% confidence interval is given in Table 3 for the sample size 150. While the method which overall provides the smallest empirical bias is GEE (but with larger standard errors for this bias leading to some confidence intervals containing 0), logistic regression provides slightly more biased results than GH estimates. This difference is marginal for small percentages of twins but increases with percentage of twins as the bias for GH decreases. Other than some case for GEE with sample size 150, none of the confidence intervals contained 0 (data not shown for sample size of 500 and 1000).

**Table 3:** Empirical bias (×10^−4^) for birthweight and 95% confidence interval for sample size 150

		Logistic regression		GLMM PQL		GLMM GH		GEE	
Percent.	Random Int.	Empirical	95% Conf	Empirical	95% Conf	Empirical	95% Conf	Empirical	95% Conf
Twins	variance	bias	Interv.	bias	Interv.	bias	Interv.	bias	Interv.
Outcome *Death* 4 variables									
0.02	2.00	9.60	[9.30 ;10.00]	-4.30	[-7.30 ; -1.30]	7.10	[6.60 ; 7.60]	0.00	[-0.60 ; 0.60]
0.05	2.00	9.90	[9.60 ; 10.30]	-45.90	[ -50.10 ; -41.70]	4.60	[4.00 ; 5.20]	0.30	[-0.20 ; 0.80]
0.10	2.00	9.90	[9.60 ; 10.30]	-44.30	[ -48.40 ; -40.20]	0.90	[ 0.20 ; 1.70]	0.50	[0.20 ; 0.90]
0.20	2.00	9.80	[9.50 ; 10.20]	-33.20	[-36.90 ; -29.50]	-1.20	[-1.90 ; -0.40]	0.40	[0.10 ; 0.80]
0.02	1.00	5.60	[5.30 ; 6.00]	-11.90	[-15.30 ; -8.50]	2.20	[1.60 ; 2.70]	0.10	[-0.20 ; 0.50]
0.05	1.00	5.60	[5.20 ; 6.00]	-60.80	[-65.90 ; -55.80]	-0.70	[-1.40 ; -0.00]	0.10	[-0.30 ; 0.50]
0.10	1.00	5.40	[5.00 ; 5.70]	-61.40	[ -66.40 ; -56.30]	-4.10	[-4.80 ; -3.30]	-0.20	[-0.50 ; 0.20]
0.20	1.00	5.60	[5.20 ; 6.00]	-47.20	[ -51.90 ; -42.40]	-5.30	[-6.10 ; -4.50]	-0.00	[-0.40 ; 0.30]
0.02	0.50	2.80	[2.50 ; 3.20]	-14.30	[-17.90 ; -10.70]	-1.80	[-2.40 ; -1.10]	0.00	[-0.50 ; 0.50]
0.05	0.50	2.70	[2.30 ; 3.10]	-65.50	[-71.00 ; -60.10]	-4.80	[-5.60 ; -4.10]	-0.20	[-0.60 ; 0.10]
0.10	0.50	2.60	[2.20 ; 2.90]	-64.00	[-69.30 ; -58.80]	-6.90	[-7.80 ; -6.10]	-0.50	[-0.90 ; -0.10]
0.20	0.50	2.60	[2.20 ; 2.90]	-48.90	[-53.80 ; -43.90]	-7.60	[-8.50 ; -6.80]	-0.50	[-0.90 ; -0.20]
Outcome *Death 2* variables									
0.02	2.00	9.60	[9.30 ; 10.00]	-4.30	[-7.30 ; -1.30]	7.10	[6.60 ; 7.60]	0.00	[-0.60 ; 0.60]
0.05	2.00	9.90	[9.60 ; 10.30]	-45.90	[-50.10 ; -41.70]	4.60	[4.00 ; 5.20]	0.30	[-0.20 ; 0.80]
0.10	2.00	9.90	[9.60 ; 10.30]	-44.30	[-48.40 ; -40.20]	0.90	[0.20 ; 1.70]	0.50	[0.20 ; 0.90]
0.20	2.00	9.80	[9.50 ; 10.20]	-33.20	[-36.90 ; -29.50]	-1.20	[-1.90 ; -0.40]	0.40	[0.10 ; 0.80]
0.02	1.00	5.60	[5.30 ; 6.00]	-11.90	[-15.30 ; -8.50]	2.20	[1.60 ; 2.70]	0.10	[-0.20 ; 0.50]
0.05	1.00	5.60	[5.20 ; 6.00]	-60.80	[-65.90 ; -55.80]	-0.70	[-1.40 ; -0.00]	0.10	[-0.30 ; 0.50]
0.10	1.00	5.40	[5.00 ; 5.70]	-61.40	[-66.40 ; -56.30]	-4.10	[-4.80 ; -3.30]	-0.20	[-0.50 ; 0.20]
0.20	1.00	5.60	[5.20 ; 6.00]	-47.20	[-51.90 ; -42.40]	-5.30	[-6.10 ; -4.50]	-0.00	[-0.40 ; 0.30]
0.02	0.50	2.80	[2.50 ; 3.20]	-14.30	[-17.90 ; -10.70]	-1.80	[-2.40 ; -1.10]	0.00	[-0.50 ; 0.50]
0.05	0.50	2.70	[2.30 ; 3.10]	-65.50	[-71.00 ; -60.10]	-4.80	[-5.60 ; -4.10]	-0.20	[-0.60 ; 0.10]
0.10	0.50	2.60	[2.20 ; 2.90]	-64.00	[-69.30 ; -58.80]	-6.90	[-7.80 ; -6.10]	-0.50	[-0.90 ; -0.10]
0.20	0.50	2.60	[2.20 ; 2.90]	-48.90	[-53.80 ; -43.90]	-7.60	[-8.50 ; -6.80]	-0.50	[-0.90 ; -0.20]
Outcome *O2 Dep.* 4 variables									
0.02	2.00	9.60	[9.30 ; 10.00]	-4.30	[-7.30 ; -1.30]	7.10	[6.60 ; 7.60]	0.00	[-0.60 ; 0.60]
0.05	2.00	9.90	[9.60 ; 10.30]	-45.90	[-50.10 ; -41.70]	4.60	[4.00 ; 5.20]	0.30	[-0.20 ; 0.80]
0.10	2.00	9.90	[9.60 ; 10.30]	-44.30	[-48.40 ; -40.20]	0.90	[0.20 ; 1.70]	0.50	[0.20 ; 0.90]
0.20	2.00	9.80	[9.50 ; 10.20]	-33.20	[-36.90 ; -29.50]	-1.20	[-1.90 ; -0.40]	0.40	[0.10 ; 0.80]
0.02	1.00	5.60	[5.30 ; 6.00]	-11.90	[-15.30 ; -8.50]	2.20	[1.60 ; 2.70]	0.10	[-0.20 ; 0.50]
0.05	1.00	5.60	[5.20 ; 6.00]	-60.80	[-65.90 ; -55.80]	-0.70	[-1.40 ; -0.00]	0.10	[-0.30 ; 0.50]
0.10	1.00	5.40	[5.00 ; 5.70]	-61.40	[-66.40 ; -56.30]	-4.10	[-4.80 ; -3.30]	-0.20	[-0.50 ; 0.20]
0.20	1.00	5.60	[5.20 ; 6.00]	-47.20	[-51.90 ; -42.40]	-5.30	[-6.10 ; -4.50]	-0.00	[-0.40 ; 0.30]
0.02	0.50	2.80	[2.50 ; 3.20]	-14.30	[-17.90 ; -10.70]	-1.80	[-2.40 ; -1.10]	0.00	[-0.50 ; 0.50]
0.05	0.50	2.70	[2.30 ; 3.10]	-65.50	[-71.00 ; -60.10]	-4.80	[-5.60 ; -4.10]	-0.20	[-0.60 ; 0.10]
0.10	0.50	2.60	[2.20 ; 2.90]	-64.00	[-69.30 ; -58.80]	-6.90	[-7.80 ; -6.10]	-0.50	[-0.90 ; -0.10]
0.20	0.50	2.60	[2.20 ; 2.90]	-48.90	[-53.80 ; -43.90]	-7.60	[-8.50 ; -6.80]	-0.50	[-0.90 ; -0.20]
Outcome *O2 Dep.* 2 variables									
0.02	2.00	9.60	[9.30 ;10.00]	-4.30	[-7.30 ; -1.30]	7.10	[6.60 ; 7.60]	0.00	[-0.60 ; 0.60]
0.05	2.00	9.90	[9.60 ; 10.30]	-45.90	[-50.10 ; -41.70]	4.60	[4.00 ; 5.20]	0.30	[-0.20 ; 0.80]
0.10	2.00	9.90	[9.60 ; 10.30]	-44.30	[-48.40 ; -40.20]	0.90	[0.20 ; 1.70]	0.50	[0.20 ; 0.90]
0.20	2.00	9.80	[9.50 ; 10.20]	-33.20	[-36.90 ; -29.50]	-1.20	[-1.90 ; -0.40]	0.40	[0.10 ; 0.80]
0.02	1.00	5.60	[5.30 ; 6.00]	-11.90	[-15.30 ; -8.50]	2.20	[1.60 ; 2.70]	0.10	[-0.20 ; 0.50]
0.05	1.00	5.60	[5.20 ; 6.00]	-60.80	[-65.90 ; -55.80]	-0.70	[-1.40 ; -0.00]	0.10	[-0.30 ; 0.50]
0.10	1.00	5.40	[5.00 ; 5.70]	-61.40	[-66.40 ; -56.30]	-4.10	[-4.80 ; -3.30]	-0.20	[-0.50 ; 0.20]
0.20	1.00	5.60	[5.20 ; 6.00]	-47.20	[-51.90 ; -42.40]	-5.30	[-6.10 ; -4.50]	-0.00	[-0.40 ; 0.30]
0.02	0.50	2.80	[2.50 ; 3.20]	-14.30	[-17.90 ; -10.70]	-1.80	[-2.40 ; -1.10]	0.00	[-0.50 ; 0.50]
0.05	0.50	2.70	[2.30 ; 3.10]	-65.50	[-71.00 ; -60.10]	-4.80	[-5.60 ; -4.10]	-0.20	[-0.60 ; 0.10]
0.10	0.50	2.60	[2.20 ; 2.90]	-64.00	[-69.30 ; -58.80]	-6.90	[-7.80 ; -6.10]	-0.50	[-0.90 ; -0.10]
0.20	0.50	2.60	[2.20 ; 2.90]	-48.90	[-53.80 ; -43.90]	-7.60	[-8.50 ; -6.80]	-0.50	[-0.90 ; -0.20]

When the variance of the intercept increases the same pattern of increased bias for logistic regression and logistic random intercept methods can be observed (data not shown).

### Coverage of the 95% confidence interval

For most scenarios, there is predominantly an over-coverage of the 95% confidence interval apart for the PQL method and the GEE method if there are only two variables. The mean coverages over all sample sizes are provided in Table 4. Over-coverage indicates that the results are conservative with increase Type II error (here the true value is not zero) [19]. For GEE with two variable, the coverage is very low indicating that the standard errors are two small.

**Table 4:** Mean coverage of the 95% confidence interval by the true value over the three sample sizes for outcome *Birthweight*

	*Death 4 covar.*			*Death 2 covar.*			*O2 dep. 4 covar*			*O2 dep 2 covar*		
Perc.	Random inter. var			Random inter. var			Random inter. var			Random inter. var		
Twins	0.5	1	2	0.5	1	2	0.5	1	2	0.5	1	2
Logistic regression												
2%	0.99	1.00	1.00	0.93	0.92	0.91	1.00	1.00	1.00	0.99	0.99	0.99
5%	0.99	1.00	1.00	0.95	0.94	0.94	1.00	1.00	1.00	0.99	0.99	0.99
10%	0.99	1.00	1.00	0.98	0.98	0.98	1.00	1.00	1.00	1.00	1.00	1.00
20%	0.99	1.00	1.00	0.99	0.98	0.98	1.00	1.00	1.00	1.00	1.00	1.00
Mixed logistic regression, PQL												
2%	0.67	0.66	0.68	0.79	0.79	0.82	0.69	0.71	0.76	0.95	0.96	0.97
5%	0.59	0.58	0.58	0.91	0.91	0.92	0.76	0.79	0.85	1.00	1.00	0.99
10%	0.57	0.56	0.58	0.96	0.96	0.97	0.87	0.89	0.93	1.00	1.00	1.00
20%	0.56	0.55	0.60	0.99	0.99	0.99	0.95	0.96	0.97	1.00	1.00	1.00
Mixed logistic regression, Gauss-Hermite												
2%	0.99	1.00	1.00	0.94	0.96	0.97	1.00	1.00	1.00	0.99	1.00	1.00
5%	0.98	0.99	0.99	0.94	0.96	0.98	1.00	1.00	1.00	0.99	1.00	1.00
10%	0.98	0.98	0.99	0.94	0.97	0.99	1.00	1.00	1.00	0.99	1.00	1.00
20%	0.97	0.97	0.98	0.95	0.98	0.99	1.00	1.00	1.00	0.99	1.00	1.00
GEE												
2%	0.98	0.98	0.98	0.50	0.56	0.60	0.99	0.99	0.99	0.59	0.67	0.77
5%	0.98	0.98	0.98	0.54	0.63	0.68	1.00	1.00	1.00	0.66	0.76	0.86
10%	0.98	0.98	0.98	0.58	0.71	0.84	1.00	1.00	1.00	0.71	0.83	0.93
20%	0.98	0.98	0.98	0.65	0.80	0.92	1.00	1.00	1.00	0.78	0.90	0.97

### Mean squared error (MSE)

**Fig. 3:**
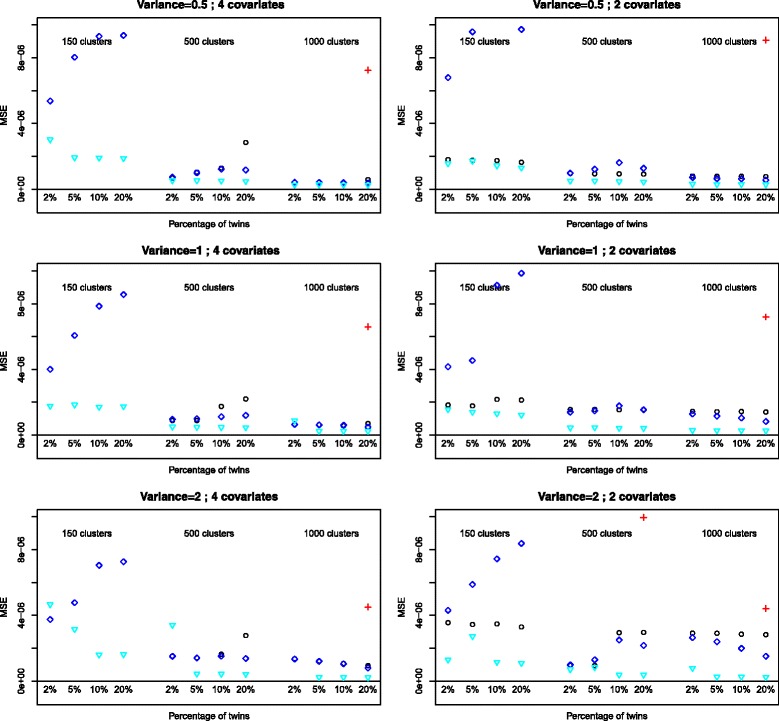
Mean squared error for the variable *Birthweight*, outcome *death*. Logistic: ∘, random intercept, PQL: +, random intercept Gauss-Hermite: ♢, GEE: $\triangledown $

**Fig. 4:**
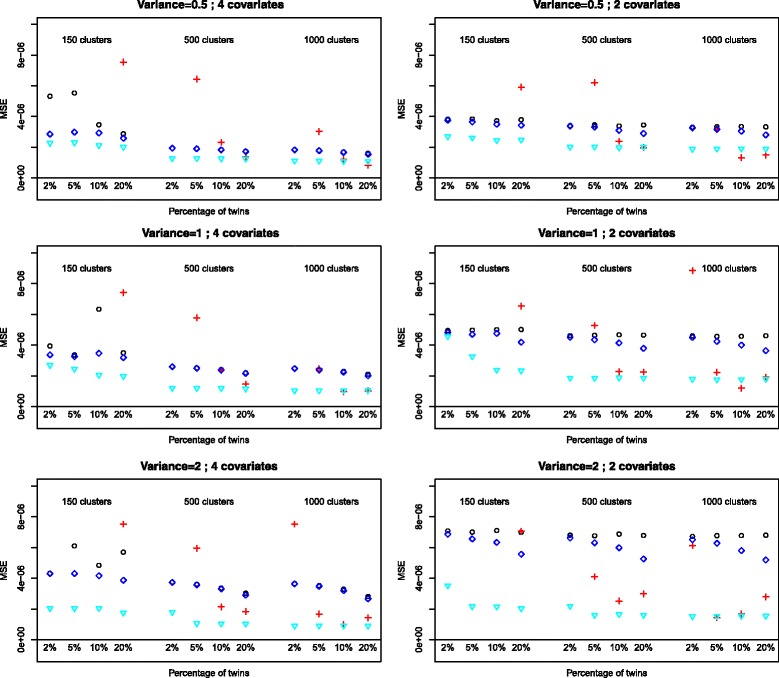
Mean squared error for the variable *Birthweight*, outcome *O*_2_ *dependence at 36 weeks*. Logistic: ∘, random intercept, PQL: +, random intercept Gauss-Hermite: ♢, GEE: $\triangledown $

GEE is the method which consistently provides the smallest mean squared error (Figs. 3 and 4). For larger sample sizes the difference with logistic regression and GH can be marginal however. GEE is also the method which is the least affected by the actual percentage of twins. The random intercept variance affects the MSE for all methods (increasing MSE with increasing variance).

### Estimation of the random variance

The logistic random intercept method of estimations GH fails to adequately estimate the variance of the random intercept if the number of clusters with two elements is too small. This might explain why the bias of estimates is similar to logistic regression when the number of clusters of size two is smaller. The mean variance estimations for the GH method are presented in Table 5.

**Table 5:** Estimated random intercept variance from mixed logistic regression model (Gauss-Hermite estimation)

	*Death 4 covar.*			*Death 2 covar.*		
Perc. Twins	Random inter. var			Random inter. var		
	0.5	1	2	0.5	1	2
2%	0.60	0.45	0.32	0.69	0.60	0.36
	(2.54)	(2.05)	(1.39)	(2.85)	(0.74)	(1.92)
5%	0.96	0.84	0.79	1.01	0.73	0.76
	(3.21)	(2.78)	(2.46)	(3.45)	(1.27)	(2.90)
10%	1.25	1.33	1.39	1.24	1.24	1.20
	(3.73)	(3.75)	(3.47)	(3.85)	(3.65)	(3.27)
20%	1.37	1.58	1.86	1.19	1.50	1.84
	(3.55)	(3.58)	(3.69)	(3.33)	(3.53)	(3.67)
	*O2 dep. 4 covar*			*O2 dep 2 covar*		
2%	0.08	0.08	0.10	0.06	0.07	0.08
	(0.23)	(0.10)	(0.10)	(0.08)	(0.08)	(0.09)
5%	0.15	0.16	0.21	0.10	0.13	0.17
	(0.44)	(0.31)	(0.31)	(0.16)	(0.19)	(0.20)
10%	0.19	0.26	0.36	0.14	0.21	0.30
	(0.43)	(0.46)	(0.46)	(0.24)	(0.35)	(0.34)
20%	0.23	0.37	0.59	0.20	0.32	0.53
	(0.34)	(0.45)	(0.54)	(0.25)	(0.32)	(0.43)

Mean value over all sample sizes (Standard deviation)

## Discussion

### The analysis of dataset of preterm infants

Our review showed that datasets with preterm infants may contain percentages of multiple birth between 10 and 30%. It seems that in most cases researchers take no particular statistical precaution to allow for the non-independence of twins in a regression model. In our small survey half the studies which did correct for clustering used a robust estimate for the standard error. Our simulation suggested that it might be an adequate solution as a simple logistic regression will provide underestimated standard errors. The two outcomes considered in this work are true binomial outcomes but researchers need to keep in mind that when a binomial outcome is in reality a continuous outcome which has been dichtotomised, then there are other issues than those encountered here. Indeed much information is lost in the process and it is easier to fit a mixed model on continuous outcomes than on binomial outcomes. If suitable, alternative methods for dichotomisation should be considered [20, 21].

The Gauss-Hermite quadrature method [22] for generalised linear mixed models has been introduced as an alternative to the penalized quasi-likelihood which have been shown to provide biased estimates. The Laplacian method is similar to the Gauss-Hermite but uses less interpolation points and therefore is quicker to converge. However these methods are known to have difficulties converging and it was not known how they would behave in cases with a limited number of very small clusters of size 2 while the rest of the data are independent. Our simulations have shown that the convergence situation will not be determined by factors like the percentage of small clusters or the number of covariates alone. However in the majority of the scenarios we simulated, the GH method had no problem converging even for the smallest sample size. In practice it will be difficult to know at the time of planning a study if the model will converge. It is therefore wise to plan an alternative in case of non-convergence. While GEE models have performed well in our simulations they can be an alternative only if population averages are of interest for the research questions.

### Reliability of estimates and coverage of 95% confidence interval

The choice of a method of analysis for data with a small number of clusters of size two depends on the purpose of the analysis. But if the aim is to compare two groups and, as for an randomised control trial, test hypotheses based for example on *p*-values, i.e significance of the test, then both reliable estimates and standard error are important. We have see that in most cases there is an over-coverage of the 95% confidence interval indicating that the results are conservatif appart for PQL and GEE if the number of covariate is two.

In the case of logistic regression, parameters will be underestimated as well as for GH methods for logistic random intercept models, though to a lesser extent especially when the number of twins increases. The results of a statistical test and the coverage of a confidence interval depend also on the bias of the parameter estimates and this should be taken into account as well as the precision of the standard error.

The similarity of results between logistic random intercept (GH method) and logistic models for bias of estimates could be explained by the inability of the GH method to correctly estimate the variance component of the model and therefore they behave like logistic regression. As the estimation of the variance component improves with increasing percentage of twins, then the underestimation of parameters which remains constant for the logistic regression, is reduced for the logistic random intercept model. As the random intercept model tends to underestimate the true variability, it may provide misleading results in terms of precision.

### Strength, limitation and further work

The strength of this study is that we based our simulation scenarios on a real dataset and considered two separate outcomes that are commonly used in neonatal research. We varied our scenarios so to evaluate how much the results obtained were dependent on the percentage of twins, sample size, variance, etc. The range of variance simulated reflected the range we saw within the two outcomes we simulated (see Table 1).

However we only tested methods of estimation implemented in R. The bias for the estimates for GEE were obtained using an approximative formula and not the GEE estimates form the real data. Doing so may have given slightly different results for bias estimates. The results are based on a single dataset with two different outcomes and on models with a limited number of covariates. This was due to the restricted availability of statistically significant covariates in the dataset. However there was no evidence of negative effects on the reliability of any of the models when increasing the number of covariates from two to four. The effect of fitting a less parsimonious model remain unknown. There are indications that the probability of the outcome may have an effect on the accuracy of the estimates for the logistic regression and logistic random intercept methods. This was not explicitly tested in this work and further research should be done to assess this.

Other methods of estimation could be investigated like the stochastic approximation expectation maximisation algorithm [23]. The MCMC method of estimation for the random intercept model has not been used in this work due to the difficulties of checking the convergence of the algorithm in an automated way, which would have been suitable for a large number of simulated datasets. However this alternative method of estimation could be considered for the analysis of data with small clusters.

### Conclusion

Our simulation study has shown that there is no single best method for the analysis of binomial outcomes with a restricted number of clusters of size two. It is a question of balance between the method offering the least bias in parameter estimates and the best measure of precision for these estimates. The GEE method seems in that respect to be safest in all situations. However there are indications, when they converge, that for larger percentage of twins, estimates are far less biased with the GH method of estimation for logistic random intercept with standard errors being acceptable. Therefore we recommend the following:

- Overall GEE method may be a reliable choice but provides population average effects;
- If the percentage of twins is large (above 10%) then the random intercept model with Gauss-Hermite method of estimation will be more reliable;
- If the logistic random intercept model does not converge even with a large percentage of twins then one could try to modify the starting value for the estimating algorithm or use either a logistic regression which will provide underestimated effects with small standard errors or use GEE.
